# Defects of microtubule cytoskeletal organization in NOA human testes

**DOI:** 10.1186/s12958-022-01026-w

**Published:** 2022-11-03

**Authors:** Xiaolong Wu, Damin Yun, Mengmeng Sang, Jianpeng Liu, Liwei Zhou, Jie Shi, Lingling Wang, Tiao Bu, Linxi Li, YingYing Huang, Dengfeng Lin, Fei Sun, C. Yan Cheng

**Affiliations:** 1 Department of Urology and Andrology, Sir Run Run Shaw Hospital, Zhejiang University School of Medicine, Hangzhou, 310016 Zhejiang China; 2 Institute of Reproductive Medicine, Nantong University School of Medicine, Nantong, 226001 Jiangsu China; 3 Department of Pathology, The First Affiliated Hospital of Wenzhou Medical University, Wenzhou, 325027 Zhejiang China; 4 The Second Affiliated Hospital of Wenzhou Medical University, Wenzhou, 325027 Zhejiang China; 5 Center for Biomedical Research, The Mary M. Wohlford Laboratory for Male Contraceptive Research, Population Council, 1230 York Ave, New York, NY 10065 USA

**Keywords:** Human testes, NOA, Microtubule cytoskeleton, MAPs, scRNA-Seq, Transcriptome profiling

## Abstract

The importance of actin and microtubule (MT) cytoskeletons in testis function in rodents is known to some extent, but its role in the etiology of azoospermia in humans remains unexplored. Here, we examined if MT cytoskeleton was defective in NOA (non-obstructive azoospermia) testes versus normal human testes based on histopathological, immunofluorescence (IF), and scRNA-Seq transcriptome profiling. Testis biopsy samples from *n* = 6 normal men *versus n* = 3 Sertoli cell only (SCO) and *n* = 3 MA (meiotic arrest) of NOA patients were used for histopathological analysis. IF analysis was also used to examine MT organization across the seminiferous epithelium, investigating the likely involvement of microtubule-associated proteins (MAPs). scRNA-Seq transcriptome profiling datasets from testes of 3 SCO patients *versus* 3 normal men in public domain in Gene Expression Omnibus (GEO) Sample (GSM) with identifiers were analyzed to examine relevant genes that regulate MT dynamics. NOA testes of MA and SCO patients displayed notable defects in MT organization across the epithelium with extensive truncation, mis-alignments and appeared as collapsed structures near the base of the tubules. These changes are in contrast to MTs in testes of normal men. scRNA-Seq analyses revealed considerable loss of spermatogenesis capacity in SCO testes of NOA patients *versus* normal men. An array of genes that support MT dynamics displayed considerable changes in expression and in spatial distribution. In summary, defects in MT cytoskeleton were noted in testes of NOA (SCO) patients, possibly mediated by defective spatial expression and/or distribution of MAPs. These changes, in turn, may impede spermatogenesis in SCO testes of NOA patients.

**Supplementary Information:**

The online version contains supplementary material available at 10.1186/s12958-022-01026-w.

## Introduction

Globally, about 15% of couples are infertile, and male factors contribute to ~ 50% of the cases [1, 2]. In these infertile men, ~ 10% are azoospermic [3, 4]. Azoospermia can be subdivided into two broad categories: obstructive azoospermia (OA) and nonobstructive azoospermia (NOA) [5–10]. Among men with azoospermia, 60% is due to OA and 40% to NOA [11]. It is known that OA is due to mechanical obstruction of the reproductive tract such as congenital bilateral absence of the vas deferens, seminal vesicle atresis, post-vasectomy, ejaculatory duct cysts/obstruction, infection-induced epididymal obstruction, spinal cord injury, diabetes or idiopathic mediated reproductive tract obstruction [9, 10, 12, 13]. For NOA, it can be the result of testicular and pretesticular defects. Testicular NOA is often the result of: (i) defects in spermatogenesis, including failure of spermatogonia to differentiate to spermatocytes, (ii) Y-chromosome disorders (e.g., microdeletion), (iii) genetic defects (e.g., Klinefelter syndrome) and (iv) infection and chemotherapy [7, 9, 10, 13]. Pre-testicular azoospermia refers to pre-testicular causes of infertility that lead to azoospermia because of extra-gonadal endocrine disorders, originating in the hypothalamus or pituitary gland. This in turn leads to deficiency of gonadotropins (e.g., FSH) or gonadotropin-releasing hormone (GnRH) secretion [7, 14, 15]. Importantly, up to 70% of NOA cases are idiopathic, which also lead to either meiotic arrest (MA) phenotype where germ cells fail to differentiate beyond primary spermatocytes, or Sertoli cell only (SCO) phenotype where tubules with complete absence of germ cells beyond spermatogonia [9, 10]. Studies have shown that SCO phenotype displays absence of spermatogonia, including spermatogonial stem cells (SSCs), due to an altered migration of primordial germ cells from yolk sac to gonadal ridges. However, in some SCO phenotypes of unknown etiology, the rare presence of spermatogonia in a small percentage of tubules are detected [9, 10, 16–18]. Additionally, recent studies using next generation sequencing [19], whole mount staining of testicular biopsy [20], and 3D-agar/methylcellulose-based culture system [21] have unequivocally demonstrated the presence of spermatogonia and SSCs in SCO testes. Nonetheless, OA and NOA can now be distinguished by FSH levels and/or testicular size based on detailed retrospective analysis of 153 azoospermic men between 1995 and 2000, including patient history, physical examination findings, endocrine profiles, testicular histology and sperm retrieval rates [22]. Specifically, of men with OA, 96% had FSH at 7.6 IU/L or less, and testis longitudinal axis greater than 4.6 cm [22]. Of men with NOA, 89% had FSH at 7.6 IU/L or greater, and testis longitudinal axis shorter than 4.6 cm [22]. This can be confirmed by testicular biopsy.

**Fig. 1:**
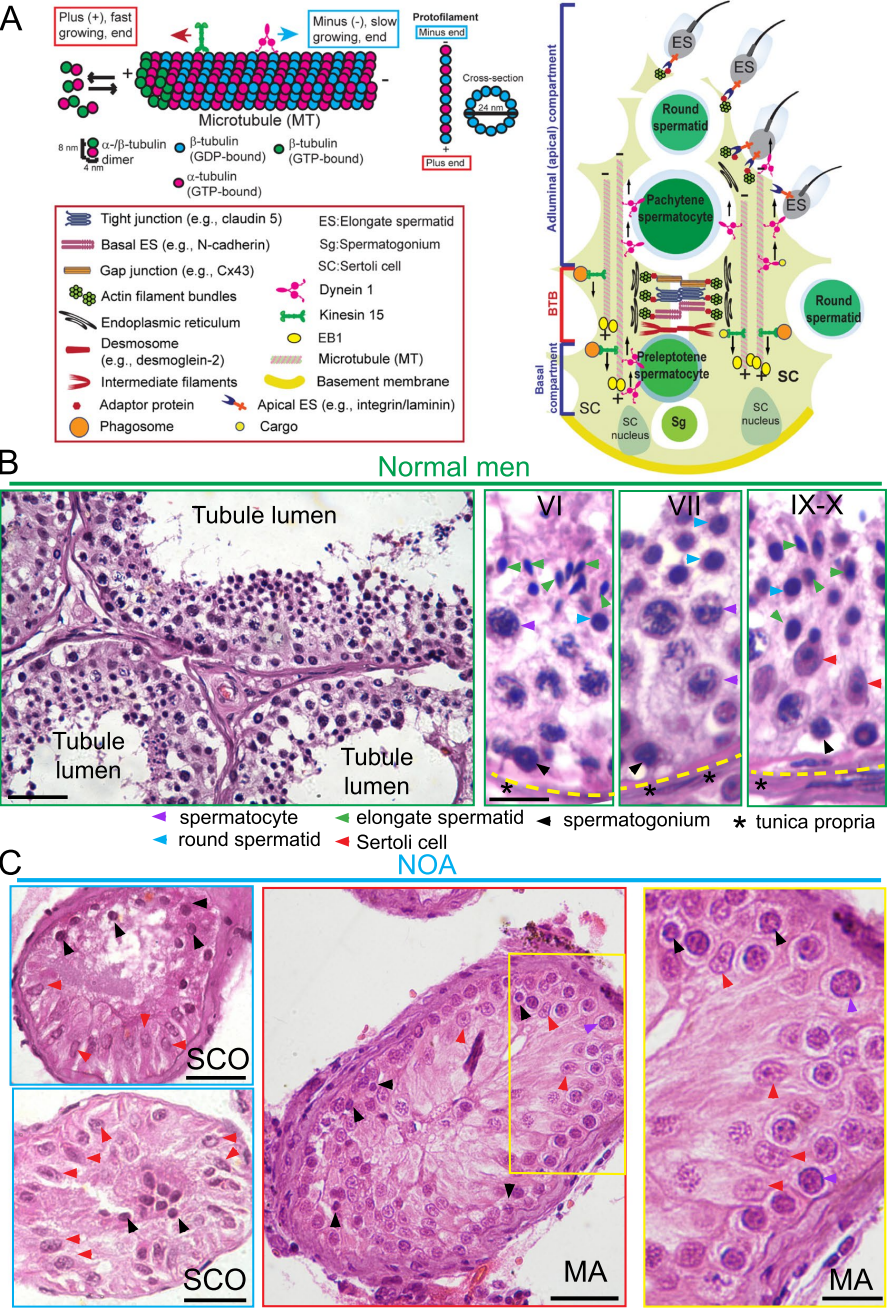
Histopathological analysis of normal vs. NOA human testes. **A** Schematic drawing on the *left* panel illustrates the polarized microtubule (MT). Each MT protofilament is formed by polymerization of GTP-bound α-/ß-tubulin heterodimers from the plus ( +), fast growing, end, that begins with the ß-tubulin, with the α-tubulin at the minus (-), flow growing, end. GTP-bound α-tubulin is non-exchangeable and never hydrolyzed. But GTP-bound ß-tubulin is rapidly hydrolyzed to become GDP-bound following assembly. A microtubule is a hollow cylindrical tube-like structure composed of 13 laterally associated protofilaments with 24-nm in diameter with an inner diameter of ~ 16 nm. The schematic drawing on the *right* panel illustrates MTs provide the structural support to Sertoli cells in the seminiferous epithelium that align perpendicular to the basement membrane of the tunica propria. MT cytoskeleton also localizes closely with the actin-based ectoplasmic specialization at the BTB known as basal ES, which is typified by the presence of actin filament bundles sandwiched between the apposing Sertoli cell plasma membranes and the endoplasmic  reticulum.  A similar ultrastructure known as apical ES is found at the Sertoli cell-elongate spermatid interface. The BTB is constituted by the actin-based TJ, basal ES and gap junction, and also intermediate filament-based desmosome, which also divides the seminiferous epithelium into the basal and adluminal (apical) compartments. The MTs serve as the tracks on which the MT-dependent motor proteins, such as dynein 1 and KIF15 (kinesin 15), move cargoes and developing spermatids to the MT minus (-) or plus ( +) end, respectively, conferring corresponding directional transport. **B** Images of cross-sections of testes from a normal human male (*left* panel) where germ cells of different types were noted. Using testes from human normal males, selected stages of tubules with different germ cell types were noted (three *right* panels). Scale bar, 200 µm (image in the *left* panel), 60 µm in the staged tubules, which apply to all other images in the three *right* panels. Keys to symbols were shown in the *lower* panel. **C** The *top* and *bottom* micrographs of the *left* panel illustrate the typical features of SCO (Sertoli cell only) testes. In the two cross-sections of SCO testes shown here, a few undifferentiated spermatogonia (black arrowheads) were routinely detected, besides Sertoli cells (red arrowheads). This observation is consistent with earlier reports, detecting few spermatogonia scattered around Sertoli cells in cross-sections of SCO testes [45–47]. In the two micrographs of the *right* panel, typical features of and MA (meiotic arrest) are noted wherein spermatocytes (purple arrowheads) failed to enter meiosis I/II to develop into haploid round spermatids and to undergo spermiogenesis to form elongate spermatids as noted in normal testes (see **B**). Scale bar, 250 µm in SCO testes encircled in blue box and in MA testes encircled in red box; and 60 µm in magnified image encircled in yellow box in the MA testis. Yellow dash line annotates location of the basement membrane in the tunica propria. Images shown herein are representative cross-sections of testes from *n* = 3 men in SCO and MA testes

Studies in rodent testes have shown that Sertoli cell cytoskeletons are crucial to support spermatogenesis and they are also the target of toxicants known to induce male reproductive dysfunction [23–28]. Microtubule (MT) and F-actin cytoskeletons are polarized structures, with the plus ( +) and minus (-) ends for the MT protofilaments *versus* the barbed ( +) and pointed (-) ends for actin filaments, located near the basement membrane and the seminiferous tubule lumen, respectively [26, 29, 30], across the seminiferous epithelium (Fig. 1A). Cytoskeletons, most notably MTs but also F-actin (but limited to stage VIII tubules [31]), which lay across the entire epithelium, aligning perpendicular to the basement membrane and serve as tracks to support transport of cellular cargoes (e.g., residual bodies, phagosomes) [26, 32] (Fig. 1A). Cytoskeletons also support the transport of developing haploid spermatids with the involvement of apical ectoplasmic specialization (ES) [25, 33, 34] (Fig. 1A). Cargo and germ cell transports are also supported by the microtubule-associated proteins (MAPs) [35]. The MAPs involved in these events are: (i) MT-dependent plus ( +) end (e.g., KIF15) and minus (-) end (e.g., dynein 1) directed motor proteins [36, 37]; and (ii) + TIPs (microtubule plus ( +) end tracking proteins, such as EB1) [38] and -TIPs (microtubule minus (-) end targeting proteins, such as CAMSAP2) [39]. Besides these MAPs, other microtubule and actin cytoskeletal proteins are also involved in germ cell transport and sperm release [40–44]. These MAPs thus serve as the crucial proteins to support intracellular protein trafficking and organelle transport (e.g., phagosome), but also germ cell transport involving ES during spermatogenesis in the rodent testis [26, 32, 34, 37, 44]. In this report, we sought to examine if there were changes in the distribution of MTs, and selected MAPs, such as EB1, dynein 1 and KIF15, across the epithelium in testes of normal *versus* NOA (including MA or SCO) men. The use of scRNA-Seq expression matrices from NOA human testes *vs.* normal men based on published GSM datasets (Tables S2) have identified additional MAPs that display considerable changes in expression in SCO testes from NOA patients *versus* normal men.

## Materials and methods

### Samples, datasets and antibodies

Sources of human biopsy testis samples (Table S1), scRNA-Seq datasets from Gene Expression Omnibus (GEO) (Table S2) were reported in Supplemental Data. Antibodies used for different experiments, including their sources, vendor information and working dilutions were summarized in Table S3.

### Collection and histopathological analysis of human testis samples

Adult human testis samples used for the experiments reported herein were approved by Nantong University Medical School (NTU) with patients’ consent for research (IRB Approved Protocol: TDLS-2020–36) entitled: “Understanding the mechanism of modification of chromatin and RNA in male infertility”. About 6-mm incision was made to the tunica albuginea of the testis of a patient in order to obtain a testis biopsy under local anesthesia. The testis biopsies taken from these patients as part of a TESE (testicular sperm extraction) which served as a therapeutic procedure to accurately diagnose the cause of infertility, including physical examination, ultrasonic testing and histological/microscopic examination by pathologists in the Nantong University Hospital Pathology Laboratory. In brief, testis biopsies were obtained from six normal adult men aged between 22 and 35 years, and six NOA patients between 28 and 34 years of age of which three were SCO and three were MA (Table S1). The healthy testis biopsy samples were from men who had no sign and/or indication of pathological conditions and infertility, who underwent emergency surgery due to testicular torsion in Emergency Room (ER) at NTU (Table S1). All samples were subjected to histological analysis following hematoxylin and eosin (H&E) staining [48] for evaluation and to define the histopathological type of NOA, including meiotic arrest (MA) and Sertoli cell only (SCO) testes, as previously described [18].

### Histological and immunofluorescence analysis (IF)

NOA patients included in this study were infertile men and the following case types were not included for histological and IF analysis: Y chromosome microdeletion and other genetic defects, chronic diseases, and hypogonadotropic hypogonadism with adjuvant hormonal therapy. Diagnosis of men with NOA, namely SCO and MA, was confirmed by physical examination and semen analysis (Table S1). Each biopsy was examined by hematoxylin and eosin (H&E) staining as described [18], and all testicular biopsies of NOA testes were divided into two categories. First, MA patients with meiosis arrest, with only Sertoli cells, spermatogonia and spermatocytes, but without detectable round and elongate spermatids in cross sections of testes (Fig. 1) due to meiotic arrest. Second, SCO patients containing Sertoli cells only found in the seminiferous epithelium, but also a few undifferentiated spermatogonia found in some cross-sections of testes intermingled with Sertoli cells (Fig. 1). These observations are consistent with earlier studies, reporting the presence of a few spermatogonia scattered around Sertoli cells in cross-sections of SCO testes [45–47]. Freshly obtained biopsy samples of human testes (Table S1) fixed immediately in Bouin’s fixative for 48 h, embedded in paraffin to obtain cross- sections of testes using a microtome, and were used for histological analysis following deparaffinization and H&E staining [48]. For IF, following deparaffinization in 100%, 95%, 70%, and 50% ethanol, cross-sections of testes were rehydrated, and washed in water thrice, 5 min each. Testis sections on slides were boiled in 10 mM sodium citrate buffer (pH 6.0 at 22°C) and then maintained in a sub-boiling temperature for 10 min for antigen retrieval. Slides cooled at room temperature for 30 min were washed thrice in PBS buffer (sodium phosphate, 0.15 M NaCl, pH 7.4 at 22°C) for 5 min each. Tissues were blocked in 5% BSA in PBS for 30 min. All tissue sections were incubated with the corresponding primary antibody (Table S3) overnight at 4°C, and then with the corresponding secondary antibodies for 1 h at room temperature. In order to confirm specificity of the staining, a negative control was used by substituting the primary antibody with the IgG obtained from the same species (e.g., normal rabbit IgG) since preimmune serum (or its IgG) of the corresponding antibody was not available commercially. To visualize cell nuclei, DAPI was used. Images were visualized and acquired using a ZEISS Imager M2 Fluorescence Microscope system with a built-in Axiocam 503 digital camera and ZEN 2 (Blue Edition), and imaging Software package (Version 3.2) (Carl Zeiss, Germany). Images were merged to examine for protein co-localization using Adobe Photoshop in Adobe Creative Suite (Version 6.0).

### scRNA-Seq and pertinent bioinformatics analysis

We selected and compared scRNA-Seq datasets obtained from Gene Expression Omnibus (GEO) Sample (GSM) with identifiers noted in Table S2, including normal men and NOA patients in the last 6 years in corresponding publications of peer-reviewed journals and listed in PubMed. The scRNA-Seq datasets of healthy humans were derived from 3 young adults of 5542 testicular cells [49]. In this report, it was focused on germ cells by examining germline-niche interactions with Leydig, myoid, Sertoli and endothelial cells, as well as macrophages, in particular the key transcriptional and epigenetic signatures to identify differences between human and mouse testes [49]. The NOA scRNA-Seq datasets were obtained from 3 SCO testes of NOA patients, and these Sertoli cells were considerably damaged based on maturation roadmap analysis [50]. In brief, we selected 3 SCO testes from NOA patients *vs.* 3 normal scRNA-Seq datasets for analysis in this report (Table S2), which were testes displaying similar pathological phenotypes as those testes used in our histopathological and IF analyses for SCO (*n* = 3) and MA (*n* = 3) testes of NOA patients (Table S1). Even though these scRNA-Seq datasets were earlier used for other analyses [49, 50], they were used herein the first time to examine the likely involvement of microtubule cytoskeleton and pertinent MT regulatory proteins in Sertoli cells on the biology of azoospermia in particular SCO testes of NOA patients. It is understood that many studies remain to be done. Nonetheless, the findings reported here serve as a roadmap for our laboratory and others for future studies based on bioinformatics data in particular the differentially expressed genes in NOA *vs.* normal testes groups pertinent to the regulation of MT cytoskeleton. In brief, scRNA-Seq datasets of human testes from healthy men (*n* = 3, 5542 cells) *versus* NOA (SCO, *n* = 3, 20,796 cells) patients were obtained by 10X Genomics using Seurat from the GEO database with corresponding Accession ID (Table S2) [49]. We used Seurat package in R toolkit (https://satijalab.org/seurat/, R package, V.3.1.2.) for analysis of the scRNA-Seq data [51] for cell type identification and clustering analysis [52], Dot plot and Violin plot analysis were used to examine differential expression (DE). First, we filtered cells according to the unique feature counts and mitochondrial counts, to be followed by using SCTransform function to normalize and scale the data. Second, we obtained cell clusters (resolution = 0.6) by UMAP (uniform manifold approximation and projection and FindNeighbors analysis) plot (and also *t*-distribute stochastic neighbor embedding, *t*-SNE, plot) and used known and published markers to identify the corresponding cell types. Third, Dot plot and Violin plot function in Seurat was used to identify the relative gene expression levels, as well as the degrees and percent expressed genes pertinent to the molecular function and biological processes of microtubule cytoskeleton in normal human testes *versus* NOA (SCO) men. The different testicular cell types were identified by Uniform Manifold Approximation and Projection (UMAP) analysis based on scRNA-Seq datasets as described [53]. We next set the parameter resolution to 0.6 for the FindClusters function to perform clustering analysis to categorize  cells in normal human and NOA (SCO) testes into different cell clusters including germ cells and somatic cells. The specific cell types, such as Sertoli cells (SC), peritubular myoid cells (PMC), Leydig cells (LC), endothelial cells (EC), testicular macrophages (tM), mast cells (MC), T cells (TC), smooth muscle cells (SM), spermatogonial stem cells (SSC), spermatogonia (SPG), spermatocytes (SPC), round spermatids (RS), elongated spermatids (ES) and sperm were confirmed by expression of corresponding marker genes to each cell type as described [53]. The marker used for SC was PRND, SOX9 [54]. The markers for PMC were MYH11 and ACTA2 [55]. The markers for LC were IGF1 [56] and DLK1 [57]. The marker for EC was VWF [53]. The markers for tM were CD68 and CD163 [58]. The marker for MC was TPSAB1 [53]. The marker for TC was CD3D [49]. The marker for SM was NOTCH3 [49]. The markers for SSC were UTF1 [59], FGFR3 [57]. The markers for SPG were TKTL1 [50] and SOHLH1 [49]. The markers for SPC were MLH3 [49], OVOL1, OVOL2 [60] and MRPL17 [49]. The markers for RS were SUN5 [49]. The markers for ES were OVOL1, SUN5, TXNDC2, TNP2, SPATA12, SPATA32 and PRM3 [49]. The markers for sperm were TNP2, SPATA12, SPATA32 and PRM3 [49]. In addition to the marker described above, we also identified other classical candidate gene markers in different cell types, such as Leydig cells (STAR, CYP11a and ID4) and spermatogonial stem cells (PAX7) to confirm the authenticity of these cells.

### RNA isolation, RT and qPCR

Total RNA was isolated from human biopsy samples using Trizol reagent (Life Technologies) and reverse-transcribed using Moloney murine leukemia virus reverse transcriptase (Promega) as described [61]. These RT products were served as templates for qPCR using corresponding primer pairs for target genes (e.g., EB1, DYNC1H1, KIF15) (Table S4). The mRNA level of a target gene was analyzed by QuantStudio™ 12 K Flex Real-Time PCR System with Power SYBR Green Master Mix (Applied Biosystems) based on the manufacturer’s Protocols with *n* = 3 independent experiments and each experiment had triplicates. GAPDH served as the internal control for normalization. Specificity of the fluorescent signal was verified by both melting curve analysis and gel electrophoresis. The expression level of a target gene was determined using 2^−ΔΔC^_T_ method.

## Results

### Histopathology of NOA testes *vs.* normal men

**Fig. 2:**
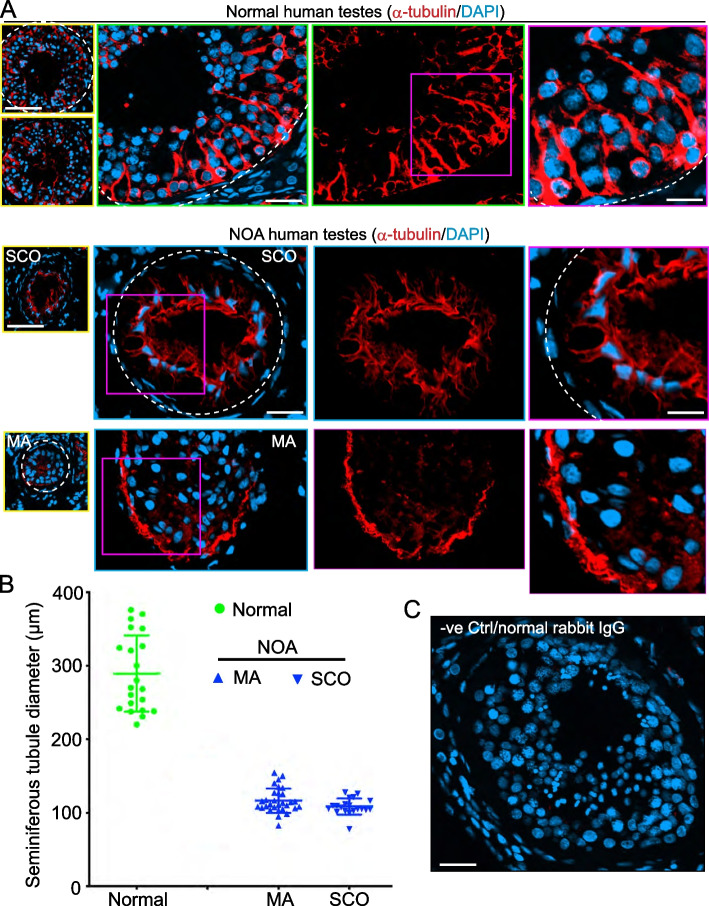
Defects in microtubule cytoskeletal organization in NOA *versus* normal human testes. **A** Microtubules (MTs) (red fluorescence, visualized by an antibody specific to α-tubulin, see Table S3), which together with ß-tubulin create the α-/ß-tubulin oligomers to serve as the building blocks of MTs. MTs appeared as track-like structures that laid perpendicular to the basement membrane (annotated by dashed white line at the base of the seminiferous epithelium as noted in normal human testes) and stretched across the entire seminiferous epithelium. Cell nuclei were visualized by DAPI (4’,6-diamidino-2-phenylindole) staining. In NOA testes, the distinctive tracks conferred by MT no longer discernible across the epithelium but considerably truncated and appeared as collapsed structures in NOA testes. As noted on the left panel where the selected tubules in low magnification were encircled in yellow, considerable reduction in tubule diameter was noted in SCO and MA testes of NOA patients compared to normal testes. Scale bar, 400 µm in the micrographs on the left panel in low magnification; 80 µm in the second panel which applies to the third panel; 40 µm in the fourth panel. **B** Changes in the seminiferous tubule diameter in randomly selected tubules from *n* = 3 men (mean ± SD) with each dot represents a tubule in normal testes (green circle) *vs.* and NOA, containing MA (meiotic arrest) and SCO (Sertoli cell only), human testes. **C** Negative (-v) control testes where primary anti-α-tubulin antibody was substituted by normal rabbit IgG and no notable red fluorescence was detected, illustrating the staining for α-tubulin noted in (**A**) was specific for MTs. Micrographs shown here were representative findings of an experiment from *n* = 3 independent experiments of testes from 3 men per group which yielded similar results

In Fig. 1A, a schematic drawing of a microtubule (MT), a polarized structure with a microtubule plus ( +), fast growing, end and a microtubule minus (-), slow growing, end. A MT protofilament is formed by polymerization of GTP-bound α-/ß-tubulin heterodimers from the plus ( +) end, that begins with the ß-tubulin, with the α-tubulin at the minus (-) end. A microtubule is a hollow cylindrical tube-like structure composed of 13 laterally associated protofilaments with a diameter of 24-nm and an inner diameter of ~ 16 nm (Fig. 1A). The schematic drawing on the *right* panel illustrates MTs provide the structural support to Sertoli cells in the seminiferous epithelium that align perpendicular to the basement membrane of the tunica propria. The MTs serve as tracks to support intracellular transport of cargoes (e.g., residual bodies, phagosomes, food vacuoles). MTs also serve as tracks to support germ cell transport, most notably developing haploid spermatids involving the testis-specific ultrastructure apical ES, during spermiogenesis, across the seminiferous epithelium [25, 30, 33, 34]. Cargo and spermatid transports are supported by the microtubule minus (-) end directed motor protein dynein 1 [36] and microtubule plus ( +) end directed motor protein kinesin 15 (KIF15) [62] (Fig. 1A). In normal adult testes of fertile men (Table S1), typical features of spermatogenesis were noted in cross sections of seminiferous tubules from these testis biopsy samples (*left* panel, Fig. 1B). The seminiferous epithelium was packed with different germ cell types, supported by Sertoli cells, and with a notable open tubule lumen in each seminiferous tubule (Fig. 1B, *left* panel). Several stages, such VI, VII, and IX-X, of the epithelial cycle were noted (Fig. 1B, in images of the corresponding right panels), which were classified using the criteria as earlier described. [63]. Each stage is comprised of several germ cell types and Sertoli cells in the seminiferous epithelium with the basement membrane located underneath. Pre-meiotic germ cells (e.g., spermatogonia, spermatocytes) and Sertoli cells were found in the basal compartment near the basement membrane with the post-meiotic germ cells (e.g., round spermatids, elongate spermatids) were in the adluminal compartment, closer to the tubule lumen (Fig. 1B). In testes of NOA, two distinctive testis phenotypes were noted, namely SCO (Sertoli cell only) and MA (meiotic arrest) tubules (Fig. 1C). In SCO, only Sertoli cells were noted across the seminiferous epithelium, and in some SCO testes, a few undifferentiated spermatogonia were found and intermingled with Sertoli cells but no spermatocytes and haploid spermatids were detected in any of the SCO testes (Fig. 1C). These findings are consistent with earlier reports, detecting few spermatogonia intermingled with Sertoli cells in the seminiferous epithelium of SCO testes [19, 45–47]. In MA testes, spermatocytes failed to differentiate into late stage spermatocytes (namely pachytene spermatocytes) to undergo meiosis to form haploid spermatids (Fig. 1C). On the other hand, both SCO and MA testes from NOA patients displayed considerably reduction in tubule diameter *vs.* normal testes, and these changes were noted in Fig. 2A and were quantified and reported in Fig. 2B.

### Defects MT cytoskeleton in NOA *vs.* normal human testes

In normal human testes, MTs were visualized by staining of α-tubulin (red fluorescence, α-tubulin together with ß-tubulin create the α-/ß-tubulin oligomers, which are the building blocks of MTs) using a specific antibody (Table S3). MTs appeared as tracks that laid perpendicular to the basement membrane (annotated by dashed white line) and stretched across the entire seminiferous epithelium (Fig. 2A, *top* panel). In cross-sections of NOA human testes, MTs in both SCO (*n* = 3) and MA (*n* = 3) testes were considerably truncated, failing to stretch across the entire seminiferous epithelium, such as noted in the MA testis or the SCO testis in Fig. 2A. Instead, MTs in SCO and MA testes appeared as a structure, collectively collapsed near the base of the epithelium in all biopsy samples examined (Fig. 2A, Table S1). In brief, MTs no longer stretched across the entire epithelium in SCO and MA testes as the tracks distinctively noted in normal control testes (Fig. 2A, *middle* and *bottom* panel *vs. top* panel). Tubule diameter was also considerably reduced, and shrunk by almost 60–70% as noted in the composite data shown in Fig. 2B. The staining of MTs as visualized by α-tubulin staining noted in Fig. 2A was specific since when the primary antibody was substituted by normal rabbit IgG (serving as a negative control due to the lack of pre-immune serum or IgG), no detectable MT staining was noted (Fig. 2C).

### Disruptive distribution of EB1 in SCO and MA testes of NOA patients

**Fig. 3:**
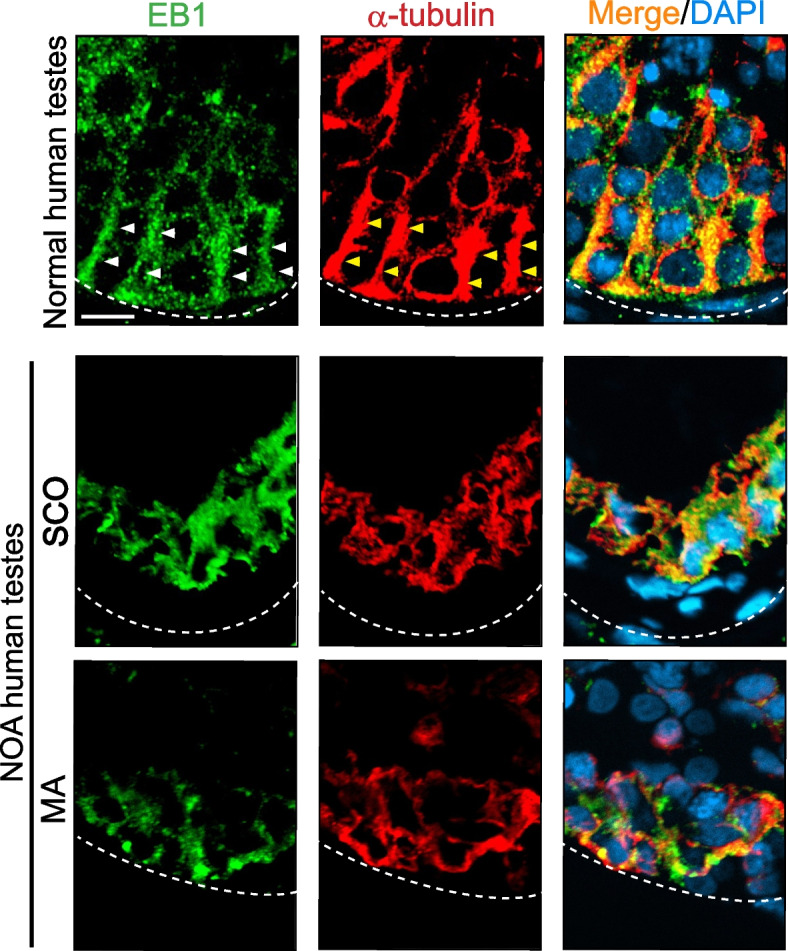
Changes in distribution of EB1 across the seminiferous epithelium in NOA *vs.* normal human testes. In cross-sections of normal human testes, EB1 (a microtubule plus ( +) end tracking protein, + TIP) appeared as aggregates (green fluorescence, see white arrow heads) that laid across the MT (visualized by α-tubulin, see yellow arrowheads) by binding to the microtubule ( +) ends to stabilize the MT protofilaments, preventing MTs from undergoing catastrophe, analogous to its distribution in the rat testis [38]. As such, EB1 also appeared as track-like structures (white arrowheads) and co-localized with MTs (visualized by α-tubulin staining, yellow arrowheads), consistent with its function to stabilize MTs [29, 64]. In NOA testes including MA and SCO testes, MTs appeared as truncated and collapsed structures, likely because EB1 no longer attached to the MT plus ( +) ends to maintain MT stabilization. Scale bar, 40 µm, which applies to all other micrographs

Studies have shown that MT end binding proteins, such as + TIPs (e.g., EB1 also known as MAPRE1, and expressed in the testis [38]), stabilize MTs, preventing MT catastrophe [64]. In normal human testes, MTs (visualized by α-tubulin, red fluorescence) appeared as tracks (yellow arrowheads) that laid across the entire seminiferous epithelium and aligned perpendicular to the basement membrane (Fig. 3, *top* panel). On the other hand, EB1 (annotated by white arrowheads), appearing as aggregates of dots located at the MT plus ( +) ends, as noted in rat testes [38], that stretched along the MT-based tracks, co-localizing exceedingly well with MTs in cross-sections of normal human testes (Fig. 3, *top, right* panel). In NOA testes, including SCO (*n* = 3) and MA (*n* = 3) testes, even though EB1 was detectable, and appeared as aggregates of dots, but EB1 no longer stretched across the entire seminiferous epithelium to serve the MT-based tracks across the seminiferous epithelium, unlike normal testes (Fig. 3). In brief, the reduced EB1 across the epithelium might affect MT dynamics, consistent with its role to maintain MT integrity in other mammalian cells and tissues [64]. It is also noted that EB1 co-localized considerably well with MTs in NOA testes (Fig. 3).

### Disruptive distribution of the MT-dependent motor proteins dynein 1 and KIF15 in SCO and MA testes

**Fig. 4:**
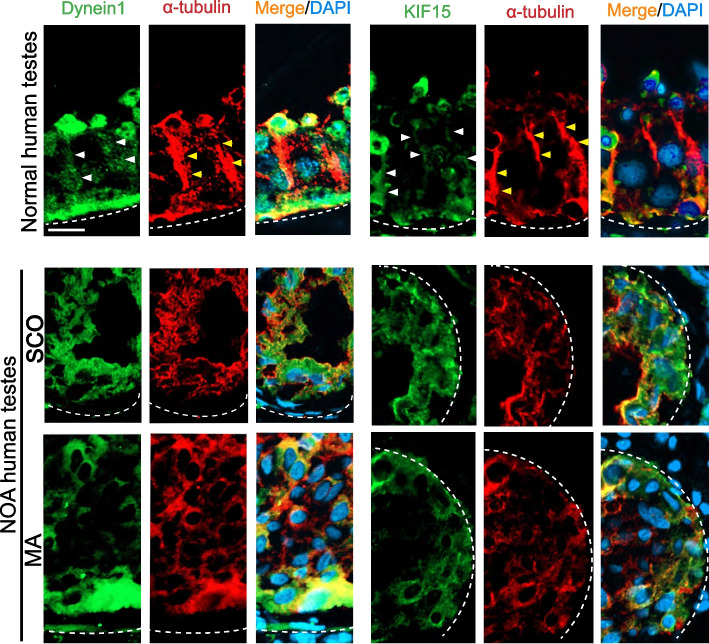
Changes in the distribution of dynein 1 and KIF15 across the seminiferous epithelium in NOA testes *vs.* normal human testes. In cross-sections of normal human testes, dynein 1 (green fluorescence, a MT minus (-) end directed motor protein) and KIF15 (green fluorescence, a MT plus ( +) end directed motor protein) which transported cargoes to the corresponding microtubule minus (-) end and plus ( +) near the tubule lumen or the basement membrane, respectively. Distribution of these motor proteins along the MT [red fluorescence, visualized by α-tubulin staining which aligned perpendicular to the basement membrane (annotated by dashed white line)] tracks was consistent with their distribution in the rat testis [35, 36]. Some aggregates of dynein 1 were also noted near the tubule lumen in the seminiferous epithelium of the stage VII tubules when spermiation just took place since dynein 1 was needed to transport and emptied these fully developed spermatids (i.e. spermatozoa) into the tubule lumen. However, in NOA (including MA and SCO) testes, dynein 1 and KIF15 no longer distributed along the MT tracks to support spermatid and other cellular transport (e.g., residual bodies, phagosomes). These changes thus impeded MT cytoskeletal organization, they were either extensively truncated and collapsed to the base of the tubule in SCO and MA testes of NOA patients. Scale bar, 40 µm, which applies to other micrographs

Dynein 1 and KIF15 are MT-dependent motor proteins that transport cellular cargoes to the minus (-) and plus ( +) end of the MT, respectively [37, 65, 66]. As such, developing haploid spermatids during spermiogenesis can be transported across the epithelium either towards the tubule lumen (in stage VI-VIII tubules) or the basement membrane (in stage V tubules) through the aid of dynein 1 or KIF15, respectively, with the critical involvement of apical ES, based on the concept earlier reported and detailed elsewhere [67]. In normal human testes, dynein 1 (green fluorescence, visualized by an anti-DYNC1H1 antibody (Table S3) that recognized the cytoplasmic dynein 1 heavy chain) (Fig. 4, *left* panel), and KIF15 (green fluorescence, visualized by an anti-IF15 that recognized the KIF15 heavy chain (Table S3) (Fig. 4, *right* panel)], appeared as aggregates of dots distributed along the MT tracks and co-localized with α-tubulin (Fig. 4). In SCO and MA testes of NOA patients, both dynein 1 and KIF15 motor proteins did not localize orderly but distributed randomly, unlike the normal testes, possibly due to the lack of the MT-based tracks (Fig. 4). These changes might impede intracellular trafficking events.

### Changes in gene expression of + TIPs in NOA *versus* normal testes in men based on scRNA-Seq analysis

**Fig. 5:**
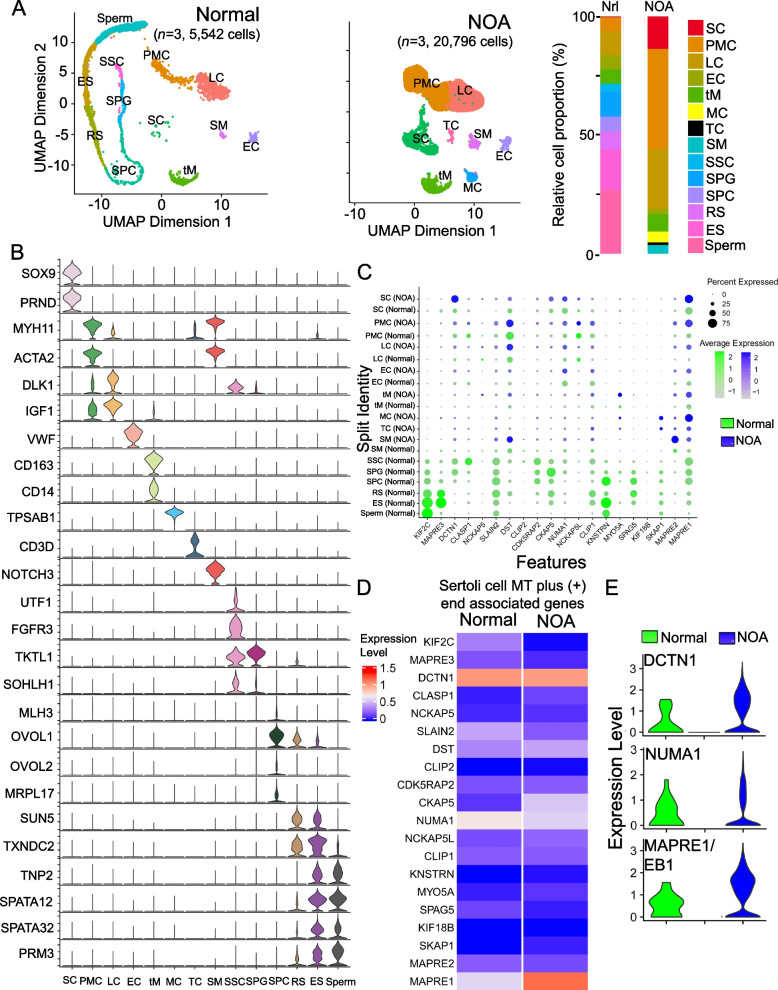
Differential expression of microtubule plus ( +) end genes that regulate microtubule dynamics in normal *vs.* NOA (SCO only) human testes. **A** t-SNE (t-distribute stochastic neighbor embedding) plot (also known as uniform manifold approximation and projection UMAP and FindNeighbors analysis) through unsupervised hierarchical clustering analysis of the scRNA-Seq datasets from human testicular cells (Table S2) to illustrate six germ cell types and six to eight somatic cell types in normal vs. NOA (SCO only) testes. Different cell types were shown with different colors as noted in the *right* panel. **B** Expression of marker genes in 14 corresponding cell types found NOA (SCO only) *vs.* normal testes. **C** Dot size represents the percent (%) expressed, and color depth represents the degree of expression level from 2 to -1. The cell types identified were Sertoli cells (SC), peritubular myoid cells (PMC), Leydig cells (LC), testicular macrophages (tM), endothelial cells (EC), mast cells, spermatogonia (SPG), round spermatids (RS), and elongated spermatids (ES). Dot size and dot color represent expression percentage and expression and expression average, respectively. **D** Heatmap of MT plus ( +) end genes in Sertoli cells displaying differential expression in normal vs. NOA (SCO only) testes. Expression level was noted by different colors. **E** Violin plots of selected MT plus ( +) end genes in Sertoli cells based on data of Dot plot **C** and Heatmap **D**, illustrating differential expression of genes in NOA (SCO only) *vs.* control testes

We next used scRNA-Seq expression matrices from Gene Expression Omnibus (GEO) of normal and NOA men (see Table S2) for analysis with the goal of identifying additional genes (or gene sets) important to support MT dynamics during spermatogenesis, besides motor proteins. Unsupervised hierarchical clustering analysis of the scRNA-Seq data illustrated by UMAP projections revealed 6 different germ cell subtypes and 6 somatic cell types including Sertoli cells in normal men with 5,542 cells were analyzed (Fig. 5A). In normal testes, 6 germ cell subtypes were noted, but mass cells (MC) and T cells (TC) were not detected besides the 6 somatic cell types with a total of 5,542 cells were analyzed (Fig. 5A). In SCO testes of NOA patients (Table S2), only 8 somatic cell types were found including mast cells (MC) and T cells (TC) without any germ cell types with a total of 20,796 cells were analyzed (Fig. 5A). UMAP plots showed that the number of ES (elongated spermatids) and sperm represented ~ 50% of the total testicular cells found in normal men, and no germ cells were found in NOA patients with SCO phenotypes in the 3 patients (Table S2). On the other hand, while mast cells (MC) and T cells (TC) were not found in normal testes, they were detected at considerable level in NOA testes. Gene markers used to identify different cell types noted in Fig. 5A were shown in Fig. 5B. Dot plot function in Seurat was used to identify the relative steady-state mRNA levels of genes pertinent to the regulation of microtubule cytoskeletal dynamics in testes that binds to the MT plus ( +) end. Studies have shown that MT dynamics are crucially regulated by end-binding proteins [26, 30, 32, 64]. For instance, EB1 (also called MAPRE1, a + TIP) is known to stabilize MTs, and we observed considerably changes in its distribution along the MTs across the seminiferous epithelium in SCO testes of NOA patents *vs.* normal testes as noted in Fig. 3. Using Dot plot analysis based on scRNA-Seq expression matrices obtained from GEO (Table S2), changes in the expression of all the known MT plus ( +) end binding proteins in all the germ cell types and somatic cell types in the testis were shown in Fig. 5C. Heatmap of genes exhibiting differential expression (DE) in Sertoli cells in SCO testes of NOA patients *vs.* normal testes were also shown in Fig. 5D. Violin plots to display the expression patterns of DCTN1, NUMA1 and MAPRE1/EB1 from normal and SCO testes of NOA patients were also shown in Fig. 5E. In brief, the expression of EB1 (i.e., MAPRE1) by Sertoli cells, which is known to promote MT stabilization in SCO/NOA testes [64], was considerably induced, when compared to normal testes based on Dot plot (Fig. 5C), Heatmap (Fig. 5D) and Violine Plot (Fig. 5E) analysis. However, since its distribution was considerably disrupted in SCO/NOA testes (Fig. 3), its increase in expression in SCO/NOA testes failed to support proper MT organization as noted in Fig. 3. These findings based on scRNA-Seq and IF shown in Fig. 3, using EB1/MAPRE1 as a candidate marker, have shown that changes in the steady-state mRNA level and the spatial distribution of a target gene/protein across the seminiferous epithelium must be taken into consideration to evaluate its role in SCO/NOA. Besides data shown in Fig. 5, Dot plots were used to depict changes in the expression of genes in all the cell types in the MT regulatory proteins responsible for microtubule organization in the testis of normal vs. SCO/NOA men (Figure S1A). Heatmap (Figure S1B) and Violin plots (Figure S1C) were also used to illustrate changes of gene expression in Sertoli cells between SCO/NOA *vs.* normal testes regarding genes pertinent to modulating MT organization. We also reported herein analyses based on Dot plots, Heatmaps and Violin plots to depict changes in gene expression in all testicular cell types and Sertoli cells pertinent to genes that promote MT bundling (Figure S2), MT nucleation (Figure S3) and MT depolymerization (Figure S4). These data thus provide helpful information to select MT regulatory genes that display notable up- or down-regulation of their steady-state mRNA levels in Sertoli cells of SCO/NOA testes for future investigation.

### Changes in gene expression of MT-dependent motor activities in NOA *vs* normal testes based on scRNA-Seq analysis

**Fig. 6:**
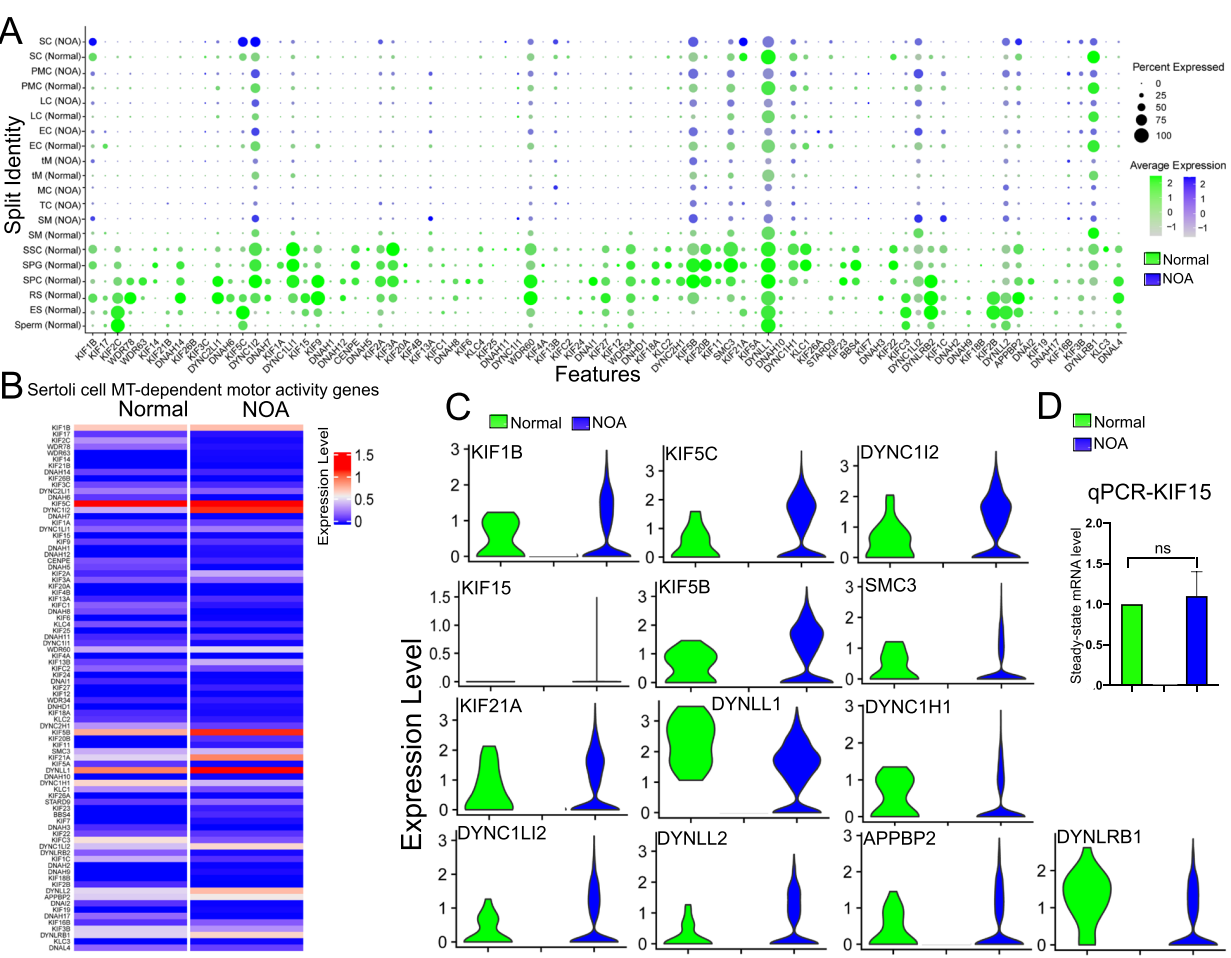
Differential expression of MT-dependent genes that modulate motor activities (including motor proteins) in normal *vs.* NOA (SCO only) human testes. **A** scRNA-Seq datasets from normal *vs.* NOA (SCO only) human testes were analyzed by Dot plot to identify differentially expressed genes encoding MT-dependent motor proteins (such as kinesins and dyneins), most notably dynein 1 (e.g., DYNC1H1, Dynein Cytoplasmic 1 Heavy Chain 1, and KIF15) as shown in Fig. 4. Dot size represents the percent (%) expressed, and color depth represents the degree of expression level from 2 to -1. The cell types were Sertoli cells (SC), peritubular myoid cells (PMC), Leydig cells (LC), endothelial cells (EC), testicular macrophages (tM), mast cells (MC), T cells (TC), smooth muscle cells (SM), spermatogonial stem cells (SSC), spermatogonia (SPG), spermatocytes (SPC), round spermatids (RS), elongated spermatids (ES) and sperm. **B** Heatmap of Sertoli cell genes that are known to modulate motor activities (including motor proteins), displaying differential expression in normal *vs.* NOA (SCO only) testes. Expression level was noted by different colors. **C** Violin plots of selected motor activity regulating genes expressed by Sertoli cells based on Dot plot (**A**) and Heatmap (**B**). **D** qPCR that compared the relative steady-state mRNA level of KIF15 in normal testes *vs.* NOA (SCO only). These data are consistent with the Dot plot (**A**), Heatmap **B**, Violin plot **C**, and IF data (Fig. 3)

Dot plot analysis based on the scRNA-Seq expression matrices have illustrated changes in the expression of MT-dependent motor activity genes in testicular cell types including somatic and germ cells in SCO/NOA *vs.* normal human testes (Fig. 6A), illustrating considerable changes in the expression of motor proteins between these groups. Heatmap (Fig. 6B) and Violin plots (Fig. 6C) were also used for differential expression (DE) analysis to identify changes in MT-dependent motor activity genes in Sertoli cells of normal *vs.* SCO/NOA testes. Two MT-dependent motor proteins, namely dynein 1 that moves cargoes to the microtubule minus (-) end, and KIF15 that moves cargoes to the microtubule plus ( +) end, as noted in Fig. 6A, have also been examined by immunofluorescence microscopy in human testes as shown in Fig. 4. The importance of dynein 1 [36] and KIF15 [62] in supporting spermatogenesis in rat testes has also been report. It is of interest to note that KIF15, when examined by qPCR, illustrating its expression in SCO/NOA *vs.* normal testes did not change considerably (Fig. 6D), consistent with data of the Dot plot (Fig. 6A), Heatmap (Fig. 6B) and Violin plot (Fig. 6C) extracted from scRNA-Seq expression matrices using datasets from Public Domain (Table S2). However, its spatial distribution across the seminiferous epithelium in SCO/NOA *vs.* normal human testes altered considerably as noted in Fig. 4. For dynein 1, it is known that genes encoding DYNLL1 (dynein light chain LC8-type 1), DYNC1H1 (dynein cytoplasmic 1 heavy chain 1) and DYNLRB1 (dynein light chain roadblock type 1) are components of the dynein 1, assembling into a highly complex functional motor protein [37, 68]. Studies by Dot blot (Fig. 6A) and Heatmap (Fig. 6B) analysis illustrated that these dynein 1 subunits were down-regulated, even though only mildly, in SCO/NOA testes. However, the immunofluorescence analysis detected striking changes in the distribution of dynein 1 (using an anti-dynein 1 heavy chain (HC), Table S3) across the seminiferous epithelium of SCO/NOA testes *vs.* normal men (Fig. 4). These changes might render this MT minus (-) end directed motor protein failed to support proper transport across the epithelium in SCO/NOA testes. More important, findings summarized in Figure S5, depicting relevant changes in expression of genes that support events pertinent to cargo transports across the seminiferous epithelium in addition to genes encoding MT-dependent motor proteins.

## Discussion

In normal human testes, microtubules (MTs) appeared as distinctive track-like structures formed by aggregates of MT protofilaments that stretched across the entire seminiferous epithelium, aligning perpendicular to the basement membrane, and noted in virtually all stages of the epithelial cycle, similar to rodent testes [26, 30, 32, 38, 69]. MTs are polarized structures in the seminiferous epithelium with the microtubule plus ( +) end and the minus (-) end located close to basement membrane and the tubule lumen, respectively [29, 30, 69] (Fig. 1A). This pattern is analogous to the cytoskeletal organization of microtubules in the rat testis [25, 30, 38, 48, 70] (Fig. 1A). Studies in other epithelia and rodent testes have shown that the tracks conferred by MTs but also F-actin serve as the “roads/highways” for “cargo-transporting vehicles”, namely the corresponding MT- or actin-based motor proteins [37]. Thus, developing germ cells and cargoes (e.g., residual bodies, phagosomes, mitochondria, food vacuoles) can be transported across the epithelium during the epithelial cycle (Fig. 1A). The directional transport of developing germ cells, most notably haploid spermatids during spermiogenesis, and other cell cargoes, are essential to support spermatogenesis. However, this directional transport mechanism relies entirely on the polarized cytoskeletons of microtubules and F-actin and the corresponding motor proteins, such as dynein 1 [36], kinesin 15 [62] and myosin VIIa [71], as noted in studies of rat testes. As noted in Fig. 1A and based on studies in this report, dynein 1 [36] and KIF15 [62] are the corresponding MT-dependent motor proteins that transport cargoes to the microtubule minus (-) and plus ( +) end, respectively. On the other hand, the MAP EB1, which is also a microtubule plus ( +) end tracking protein to promote MT stabilization also bind to the MT-based tracks as noted in the normal human testes, similar to its distribution in the rat testis [38]. Their defective distribution across the seminiferous epithelium in OA and NOA testes as reported here thus impeded proper organization of MT-based tracks to support intracellular cargo transport in the testis to maintain spermatogenesis. As noted, the MT tracks appeared to become a broken network of tracks, appearing as a collapsed structure, in NOA testes, unlike the properly organization directional network of tracks seen in normal testes. Nonetheless, an alternative, perhaps equally possible, explanation for the reported phenotypes regarding MT cytoskeleton is that since germ cells are absent in SCO/NOA testes, Sertoli cells thus lack the normal adhesion/paracrine/morphological interactions with germ cells, yet these cell–cell contact based signaling are necessary to stimulate MT dynamics in Sertoli cells. These possibilities must be carefully evaluated in future studies.

In this report, we have discovered several unexpected yet striking findings that deserve attentions and further investigations. First, the use of UMAP plots to display clusters of germ cells and somatic cells in normal *versus* SCO/NOA testes were somewhat unexpected. We did not present data on OA testes in this study since some studies have shown that OA men have normal spermatogenesis in the testis [72]. While our preliminary analysis had suggested some changes in the proportion of germ cell classes in OA *vs.* normal testes, additional testes from OA patients are needed to confirm these observations. Nonetheless, our findings reported here offer a plausible explanation for the considerable defects of spermatogenesis in NOA patients, including MA and SCO testes, as noted in Figs. 2 and 5A. For instance, extensive defects in microtubule organization across the seminiferous epithelium in SCO and MA testes of NOA patients *vs.* normal men were obviously noted (Fig. 2). These changes, in turn, could impede the seminiferous tubule diameter. It could also be possible that the overall structure, shape or polarity of both germ and Sertoli cells are different in NOA testes, thus leading to MT mis-alignment. The changes noted in Fig. 2 are likely the results of defects in spatial expression of the MT end binding proteins, such as EB1 (also known as MAPRE1) which stabilizes MT cytoskeleton, preventing MT catastrophe [73] (Fig. 3). Furthermore, defects in spatial expression of the MT-dependent motor proteins dynein 1 and KIF15, recently shown to be crucial to support MT dynamics in rodent testes [36, 62] were also detected (Fig. 4). In brief, the disruptive changes in spatial expression and distribution of these MT regulatory proteins impede MT cytoskeletal organization across the seminiferous epithelium to support the initiation of spermatogenesis (in SCO testes) and the completion of meiosis (in MA testes). This notion is also supported by findings reported in Figures S1-S5 based on limited analysis reported herein. Nonetheless, these observations require additional studies when more samples become available.

Second, notable defects in microtubule organization across the seminiferous epithelium in all testes of SCO and MA from NOA patients *vs.* normal men were detected. These defects apparently might impede intracellular trafficking of organelles (e.g., residual bodies, endocytic vesicles, cytoplasmic droplets, phagosomes, Golgi, food vacuoles) and the loss of necessary cellular scaffolds to support cellular events to sustain the epithelial cycle of spermatogenesis. It is noteworthy that that these data do not provide conclusive proof regarding causal relationship between defective MT cytoskeleton and infertility, rather they suggest changes in MTs in different conditions of spermatogenic failure. However, the multiple genes detected in SCO/NOA testes by bioinformatics analysis reported here (Figures S1-S5) have provided a roadmap for future studies to examine the involvement of MT regulatory genes and male infertility. These would include the use of genetic models in mice, and the search of genetic variants in humans. In brief, much work is needed in future studies to further confirm the role of MT cytoskeleton in NOA.

Studies in other epithelia have shown that the homeostasis of MT cytoskeleton is regulated by an array of microtubule-associated proteins (MAPs) [74], but most notably the end-binding proteins, such as + TIPs (e.g., EB1) and motor proteins (e.g., dynein 1, KIF15), which in turn provide the means to support MT dynamics by conferring intracellular trafficking. In the testis, EB1 and motor proteins have been shown to be essential to support spermatogenesis including spermatid transport. For instance, a knockdown (KD) of dynein 1 in the rat testis in vivo by RNAi was associated with defects in spermatid transport across the seminiferous epithelium since step 19 spermatids were trapped deep inside the epithelium in stage IX-XII tubules when spermiation had taken place at stage VIII [36]. Furthermore, step 19 spermatids were intermingled with step IX, X, XII and XII spermatids in the epithelium until they were phagocytosed in stage XIII-XIV tubules [75]. Furthermore, multiple defective epididymal spermatozoa were found in the epididiymis including deformed sperm heads, defective mid-piece and broken sperm tails, which were likely the results of defects in endocytic vesicle-mediated trafficking since dynein 1 is crucial to direct cargoes to the MT minus (-) end of MTs [75]. A reduction of dynein 1 in the testis by 70% following its KD thus impeded intracellular protein trafficking events necessary to support spermatid development during spermiogenesis. Nonetheless, the etiology of SCO and MA of NOA remains to be elucidated since the phenotypes noted in both types of azoospermia are the results of interactions of multiple players as noted in the different MAP genes in Dot plots reported herein based on analysis of the scRNA-Seq expression matrices of testes from SCO and MA testes of NOA patents *vs.* normal men. However, the bioinformatics findings reported here provide an important database by selecting other relevant genes and proteins, in particular MAPs and MT polymerization and depolymerization regulatory genes/proteins that display wide fluctuations between normal and SCO/MA testes of NOA patients. This information also serves as the roadmap that should be helpful to elucidate their functions in supporting MT dynamics and human spermatogenesis.

Additionally, the findings noted in Figs. 1, 2, 3, and 4 have shown that histopathological analysis of testis biopsy samples, such as monitoring MT organization, coupled with IF analysis for selected MT regulatory proteins would be of interest in future studies to elucidate the relationship of cytoskeletons and male infertility. Interestingly, genes/proteins that may not display notable changes in their expression, such as KIF15 (Fig. 6D) and a mild increase in EB1 (also known as MAPRE1) in SCO testes, between and SCO and MA testes of NOA patients *vs.* normal testes based on Dot plots, Violin plots, or Heatmaps. Yet, an examination on the alteration of their spatial expression and distribution as in NOA *vs.* normal testes may provide helpful information to study the etiology of infertility. Needless to say, these preliminary findings must be further expanded in future studies.

Herein, we have reported preliminary findings regarding the likely involvement of MT cytoskeleton in the defects noted in NOA testes, in particular through analysis of scRNA-based transcriptome profiling datasets and immunofluorescence analysis. Due to the limited sample size used in this report, these observations must be further confirmed and expanded in future investigations. Furthermore, the bioinformatics findings reported here have yielded helpful insights, which are being actively investigated in our laboratory in ongoing studies by recruiting additional patients including normal men. But they are equally helpful to other investigators. In brief, it is likely that MTs are differentially organized in the testes of normal and SCO/MA men, and their defective organization most likely affects other cellular events to support spermatogenesis such as cargo transport across the seminiferous epithelium. Additional studies should include other morphological studies such as changes in cell shapes (plasma membrane), cell polarity and others, including MT polarity. 

## Supplementary Information

**Additional file 1:** **Figure S1.** Differential expression of genes encoding proteins that regulate MT organization in normal *vs. *OA and NOA human testes based on analyses of scRNA-Seq datasets. (A) scRNA-Seq datasets from normal *vs. *OA and NOA human testes were analyzed used Seurat package in R toolkit using Rstudio to obtain Dot plot to identify differentially expressed genes encoding genes pertinent to MT organization. It is noted that some of these proteins overlap with proteins examined in Figures S2-S5. Dot size represents the percent (%) expressed, and color depth represents the degree of expression level from 2 to -1. The cell types were Sertoli cells (SC), peritubular myoid cells (PMC), Leydig cells (LC), testicular macrophages (tM), endothelial cells (EC), mast cells, spermatogonia (SPG), round spermatids (RS), and elongated spermatids (ES). (B) Heatmap that illustrate some Sertoli cell differentially regulated genes involved in MT organization in OA and NOA *vs. *normal testes. (**C**) Violin plots of some selected Sertoli cell differentially regulated genes involved in MT organization in OA and NOA *vs*. normal testes. **Figure S2.** Differential expression of genes encoding proteins involved in microtubule bundling in normal *vs. *OA and NOA human testes based on analyses of scRNA-Seq datasets. (A) Dot plot of all genes in different testicular cell types that are involved in MT bundling in normal *vs. *OA nd NOA testes. Dot size represents the percent (%) expressed, and color depth represents the degree of expression level from 2 to -1. The cell types examined herein can be found in Legend to Figure S1. These MT bundling proteins are also involved in mitosis and meiosis to support sister chromatid separation, but also unique structural function such as ectoplasmic specialization (ES) in the testis to support spermatogenesis, as well as structural MAPs (e.g., MAP1a) that confer MT stabilization. For example, MAP1b and MAP1s are involved in the formation of microtubule bundles in neurons 1, 2, 3. (B) Heatmap that illustrate some Sertoli cell differentially regulated genes involved in MT bundling in normal vs. OA and NOA testes. (C) Violin plots of selected Sertoli cell differentially regulated genes involved in MT bundling in normal vs. OA and NOA testes. **Figure S3.** Differential expression of genes encoding proteins involved in MT nucleation in normal *vs. *OA and NOA human testes based on analyses of scRNA-Seq datasets. (A) Dot plot of all genes in different testicular cell types that are involved in MT nucleation in normal *vs. *OA and NOA testes. Dot size represents the percent (%) expressed, and color depth represents the degree of expression level from 2 to -1. The cell types examined herein can be found in Legend to Figure S1. (B) Heatmap that illustrate some Sertoli cell differentially regulated genes involved in MT nucleation in normal vs. OA and NOA testes. (C) Violin plots of selected Sertoli cell differentially regulated genes involved in MT nucleation in normal vs. OA and NOA testes. **Figure S4.** Differential expression of genes encoding proteins involved in MT depolymerization in normal *vs. *OA and NOA human testes based on analyses of scRNASeq datasets. (A) Dot plot of all genes in different testicular cell types that are involved in MT depolymerization in normal *vs. *OA and NOA testes. Dot size represents the percent (%) expressed, and color depth represents the degree of expression level from 2 to -1. The cell types examined herein can be found in Legend to Figure S1. (B) Heatmap that illustrate some Sertoli cell differentially regulated genes involved in MT depolymerization in normal vs. OA and NOA testes. (C) Violin plots of selected Sertoli cell differentially regulated genes involved in MT depolymerization in normal vs. OA and NOA testes. **Figure S5.** Differential expression of genes encoding proteins involved in MT-based cargo transport in normal *vs. *OA and NOA human testes based on analyses of scRNASeq datasets. (A) Dot plot of all genes in different testicular cell types that are involved in MTbased cargo transport (e.g., spermatids, residual bodies, phagosomes) across the seminiferous epithelium in normal *vs. *OA and NOA testes. Dot size represents the percent (%) expressed, and color depth represents the degree of expression level from 2 to -1. The cell types examined herein can be found in Legend to Figure S1. (B) Heatmap that illustrate some Sertoli cell differentially regulated genes involved in MT-based cargo transport in normal vs. OA and NOA testes. (C) Violin plots of selected Sertoli cell differentially regulated genes involved in MTbased cargo transport in normal *vs. *OA and NOA testes. **Table S1.** Patient information to obtain testis biopsy samples for histopathology and immunofluorescence analysis*. **Table S2**. Sources of scRNA-Seq datasets **Table S3.** Antibodies used for different experiments in this report*. Table S4. Primers used for qPCR.

## Data Availability

Some data generated or analyzed during this study are included in this published article (e.g., image files, gel analysis data and composite data reported in Figs. 1 through 6, or in the data repositories listed in Table S2 (for scRNA-Seq datasets). These raw data, including all image (microscopy) data are available from the Corresponding Authors on reasonable request. However, all datasets used for studies reported in Figs. 1 through 6 and Supplemental Figures S1 through S5 pertinent to scRNA-Seq are publicly available as noted in Accession ID with appropriate GSM ID information, which are freely available for downloads.
