# Supplementary material for: Defects of microtubule cytoskeletal organization in NOA human testes
Source: Reprod Biol Endocrinol. 2022 Nov 3;20:154. doi: 10.1186/s12958-022-01026-w (PMC9632130; doi:10.1186/s12958-022-01026-w)

Supplementary Data

Figure S1. Differential expression of genes encoding proteins that regulate MT organization in normal *vs.* OA and NOA human testes based on analyses of scRNA-Seq datasets. (A) scRNA-Seq datasets from normal *vs.* OA and NOA human testes were analyzed used Seurat package in R toolkit using Rstudio to obtain Dot plot to identify differentially expressed genes encoding genes pertinent to MT organization. It is noted that some of these proteins overlap with proteins examined in **Figures S2-S5**. Dot size represents the percent (%) expressed, and color depth represents the degree of expression level from 2 to -1. The cell types were Sertoli cells (SC), peritubular myoid cells (PMC), Leydig cells (LC), testicular macrophages (tM), endothelial cells (EC), mast cells, spermatogonia (SPG), round spermatids (RS), and elongated spermatids (ES). **(B)** Heatmap that illustrate some Sertoli cell differentially regulated genes involved in MT organization in OA and NOA *vs.* normal testes. **(C)** Violin plots of some selected Sertoli cell differentially regulated genes involved in MT organization in OA and NOA *vs*. normal testes.

Figure S2. Differential expression of genes encoding proteins involved in microtubule bundling in normal *vs.* OA and NOA human testes based on analyses of scRNA-Seq datasets. (A) Dot plot of all genes in different testicular cell types that are involved in MT bundling in normal *vs.* OA and NOA testes. Dot size represents the percent (%) expressed, and color depth represents the degree of expression level from 2 to -1. The cell types examined herein can be found in Legend to **Figure S1**. These MT bundling proteins are also involved in mitosis and meiosis to support sister chromatid separation, but also unique structural function such as ectoplasmic specialization (ES) in the testis to support spermatogenesis, as well as structural MAPs (e.g., MAP1a) that confer MT stabilization. For example, MAP1b and MAP1s are involved in the formation of microtubule bundles in neurons [1, 2, 3]. **(B)** Heatmap that illustrate some Sertoli cell differentially regulated genes involved in MT bundling in normal vs. OA and NOA testes. **(C)** Violin plots of selected Sertoli cell differentially regulated genes involved in MT bundling in normal vs. OA and NOA testes.

Figure S3. Differential expression of genes encoding proteins involved in MT nucleation in normal *vs.* OA and NOA human testes based on analyses of scRNA-Seq datasets. (A) Dot plot of all genes in different testicular cell types that are involved in MT nucleation in normal *vs.* OA and NOA testes. Dot size represents the percent (%) expressed, and color depth represents the degree of expression level from 2 to -1. The cell types examined herein can be found in Legend to **Figure S1**. **(B)** Heatmap that illustrate some Sertoli cell differentially

regulated genes involved in MT nucleation in normal vs. OA and NOA testes. **(C)** Violin plots of selected Sertoli cell differentially regulated genes involved in MT nucleation in normal vs. OA and NOA testes.

Figure S4. Differential expression of genes encoding proteins involved in MT depolymerization in normal *vs.* OA and NOA human testes based on analyses of scRNA-Seq datasets. (A) Dot plot of all genes in different testicular cell types that are involved in MT depolymerization in normal *vs.* OA and NOA testes. Dot size represents the percent (%) expressed, and color depth represents the degree of expression level from 2 to -1. The cell types examined herein can be found in Legend to **Figure S1**. **(B)** Heatmap that illustrate some Sertoli cell differentially regulated genes involved in MT depolymerization in normal vs. OA and NOA testes. **(C)** Violin plots of selected Sertoli cell differentially regulated genes involved in MT depolymerization in normal vs. OA and NOA testes.

Figure S5. Differential expression of genes encoding proteins involved in MT-based cargo transport in normal *vs.* OA and NOA human testes based on analyses of scRNA-Seq datasets. (A) Dot plot of all genes in different testicular cell types that are involved in MT-based cargo transport (e.g., spermatids, residual bodies, phagosomes) across the seminiferous epithelium in normal *vs.* OA and NOA testes. Dot size represents the percent (%) expressed, and color depth represents the degree of expression level from 2 to -1. The cell types examined herein can be found in Legend to **Figure S1**. **(B)** Heatmap that illustrate some Sertoli cell differentially regulated genes involved in MT-based cargo transport in normal vs. OA and NOA testes. **(C)** Violin plots of selected Sertoli cell differentially regulated genes involved in MT-based cargo transport in normal *vs.* OA and NOA testes.

References

1. Orbán-Németh Z, Simader H, Badurek S, Trancikovά A, Propst F. Microtubule-associated protein 1S, a short and ubiquitously expressed member of the microtubule-associated protein 1 family. *J Biol Chem* **280**, 2257-2265 (2005).
2. Feltrin D, *et al.* Growth cone MKK7 mRNA targeting regulates MAP1b-dependent microtubule bundling to control neurite elongation. *PLoS Biol* **10**, e1001439 (2012).
3. Bodaleo FJ, Montenegro-Venegas C, Henríquez DR, Court FA, Gonzalez-Billault C. Microtubule-associated protein 1B (MAP1B)-deficient neurons show structural presynaptic deficiencies in vitro and altered presynaptic physiology. *Scientific reports* **6**, 30069 (2016).

Table S1. Patient information to obtain testis biopsy samples for histopathology and immunofluorescence analysis*

Medical condition and age		Serum hormone levels						Fertility status
**Diagnosis**	**Age**	**FSH (1.27-19.26 IU/L)**	**LH (1.24-8.62 IU/L)**	**PRL (2.64-13.13 ng/ml)**	**T (6.1-27.1 nmol/L)**	**E (<143 pmol/L)**	**Inhibin B (20.37-206.21 pg/ml)**	
Normal 1	22	Not determined						Yes
Normal 2	25							Yes
Normal 3	30							Yes
Normal 4	29							Yes
Normal 5	33							Yes
Normal 6	35							Yes
NOA 1 (MA)	28	17.4	13.6	10.84	20.56	85	20.3	No
NOA 2 (MA)	32	30.2	22.7	15.6	17.4	132	10.3	No
NOA 3 (MA)	34	25.6	19.6	12.8	16.4	98	30.2	No
NOA 4 (SCO)	32	17.3	15.3	13.6	15.9	142	30.3	No
NOA 5 (SCO)	34	23.9	25.3	14.9	15.3	84	28.5	No
NOA 6 (SCO)	29	31.4	23.5	17.4	13.4	93	15.4	No

∗ Serum hormone levels of the normal subjects were not available since these patients (normal subjects) were admitted to undergo emergency surgery due to testicular torsion. Ranges of the hormone serum levels for FSH, LH, PRL (prolactin), T (testosterone), E (estratiol-17ß) and inhibin B are based on the normal range in the clinics at Renji Hospital, Shanghai Jiaotong University School of Medicine (Shanghai, China).

Table S2. Sources of scRNA-Seq datasets

ID	Condition	Library	Reference	Accession ID
Donor 1	Healthy subject	10X Genomics	[1]	GSM3052917; GSM3052918
Donor 2	Healthy subject	10X Genomics	[1]	GSM3052919; GSM3052920
Donor 3	Healthy subject	10X Genomics	[1]	GSM3052921; GSM3052922
NOA 1	NOA (SCO only)	10X Genomics	[2]	GSM4504195
NOA 2	NOA (SCO only)	10X Genomics	[2]	GSM4504196
NOA 3	NOA (SCO only)	10X Genomics	[2]	GSM4504197

References

1. Guo J*, et al.* The adult human testis transcriptional cell atlas. *Cell research* **28**, 1141-1157 (2018).

2. Zhao L*, et al.* Single-cell analysis of developing and azoospermia human testicles reveals central role of Sertoli cells. *Nat Commun* **11**, 5683 (2020).

Table S3. Antibodies used for different experiments in this report*

Antibody	Host species	Vendor	Catalog No	Working Dilution
α-Tubulin	Mouse	Proteintech	66031-1-Ig	1:400
α-Tubulin	Rabbit	Proteintech	11224-1-AP	1:400
EB1	Mouse	BD Biosciences	610534	1:50
CAMSAP2	Rabbit	Proteintech	17880-1-AP	1:100
Phospho-S6 Ribosomal Protein (Ser235/236)	Rabbit	Cell Signaling Technology	4858s	1:50
Phospho-S6 Ribosomal Protein (Ser240/244)	Rabbit	Cell Signaling Technology	5364s	1:100
MAP1A	Rabbit	Abcam	ab101224	1:100
MARK4	Rabbit	Invitrogen	PA5-83450	1:200
DYNC1H1 (dynein 1 heavy chain)	Rabbit	Proteintech	12345-1-AP	1:200
KIF15	Rabbit	Proteintech	55407-1-AP	1:200
p-FAK-Tyr407	Rabbit	Invitrogen	44-650G	1:100

*Antibodies used for experiments reported here were shown to cross-react with the corresponding human proteins as indicated by the corresponding manufacturer.

Table S4. Primers used for qPCR

Gene	Primer sequence	**Orientation**	**Nucleotide Position**	**Tm, °C**	**GenBank Accession Number**
MAPRE1	5’-AGGCCCATCTCAACACAGAG-3’	Sense	484-504	60	
(*EB1*)	5’-CGTTCTCCTGGCAAATCAAT-3’	Antisense	694-714		
KIF15	5’-ATCGTCTAGGTCTCATGCCG-3’	Sense	696-716	59	NM_020242.3
	5’-TCCATTACCCACGTCGACAA-3’	Antisense	924-944		
DYNEIN1	5’-GGAAGTCAACGTCACCACCT-3’	Sense	13623-13643	60	NM_001376.5
	5’-TCTCGGTGTTTGTCTGCTTG-3’	Antisense	13798-13818		
ß-ACTIN	5’-GGACTTCGAGCAAGAGATGG-3’	Sense	663-683	59	NM_001101.5
	5’-AGCACTGTGTTGGCGTACAG-3’	Antisense	896-916		

Abbreviation used: Tm, annealing temperature. Primer was designed by primer3 (https://www.ncbi.nlm.nih.gov/tools/primer-blast/)

Figure S1 (Wu X et al.)

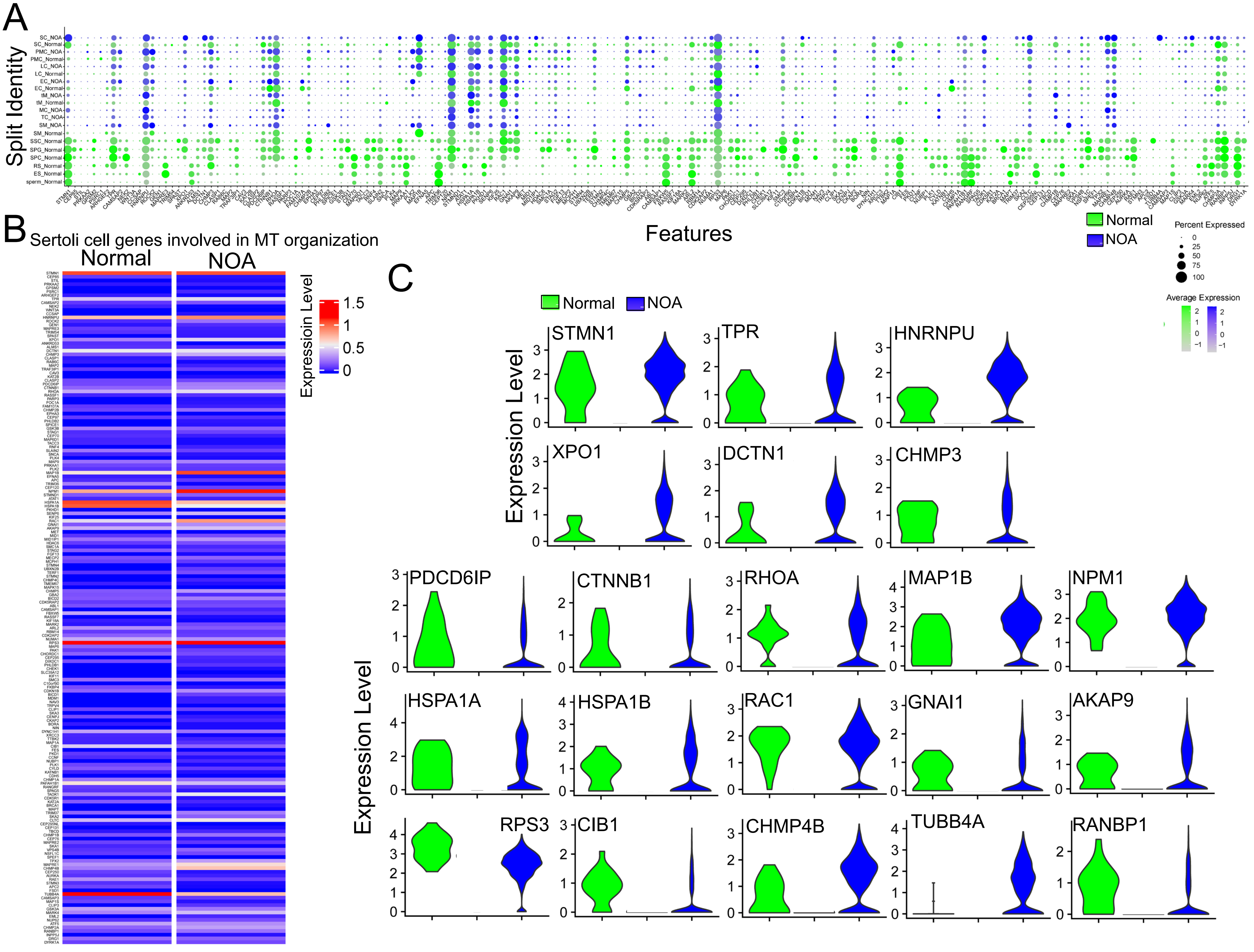

Figure S2 (Wu X et al.)

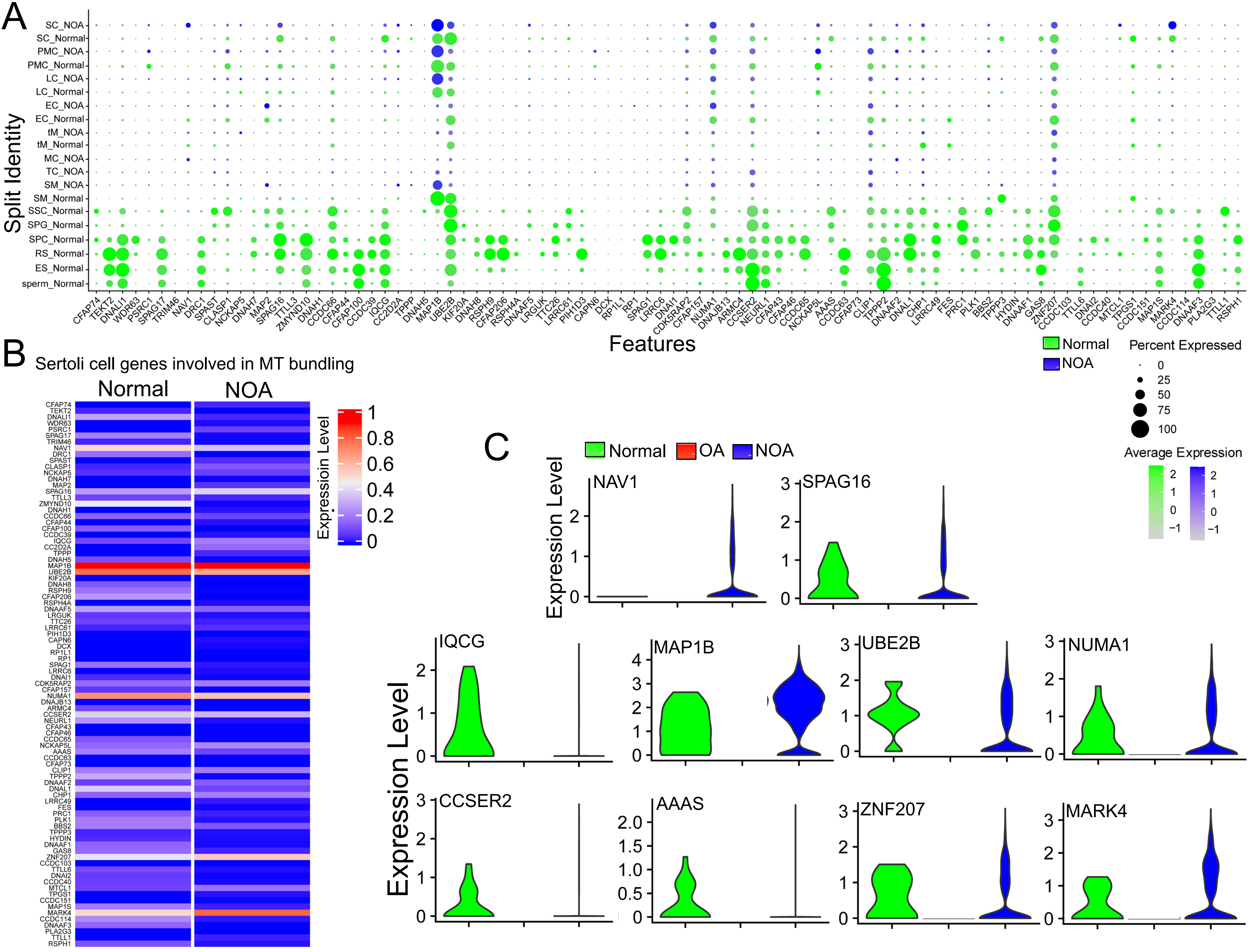

Figure S3 (Wu X et al.)

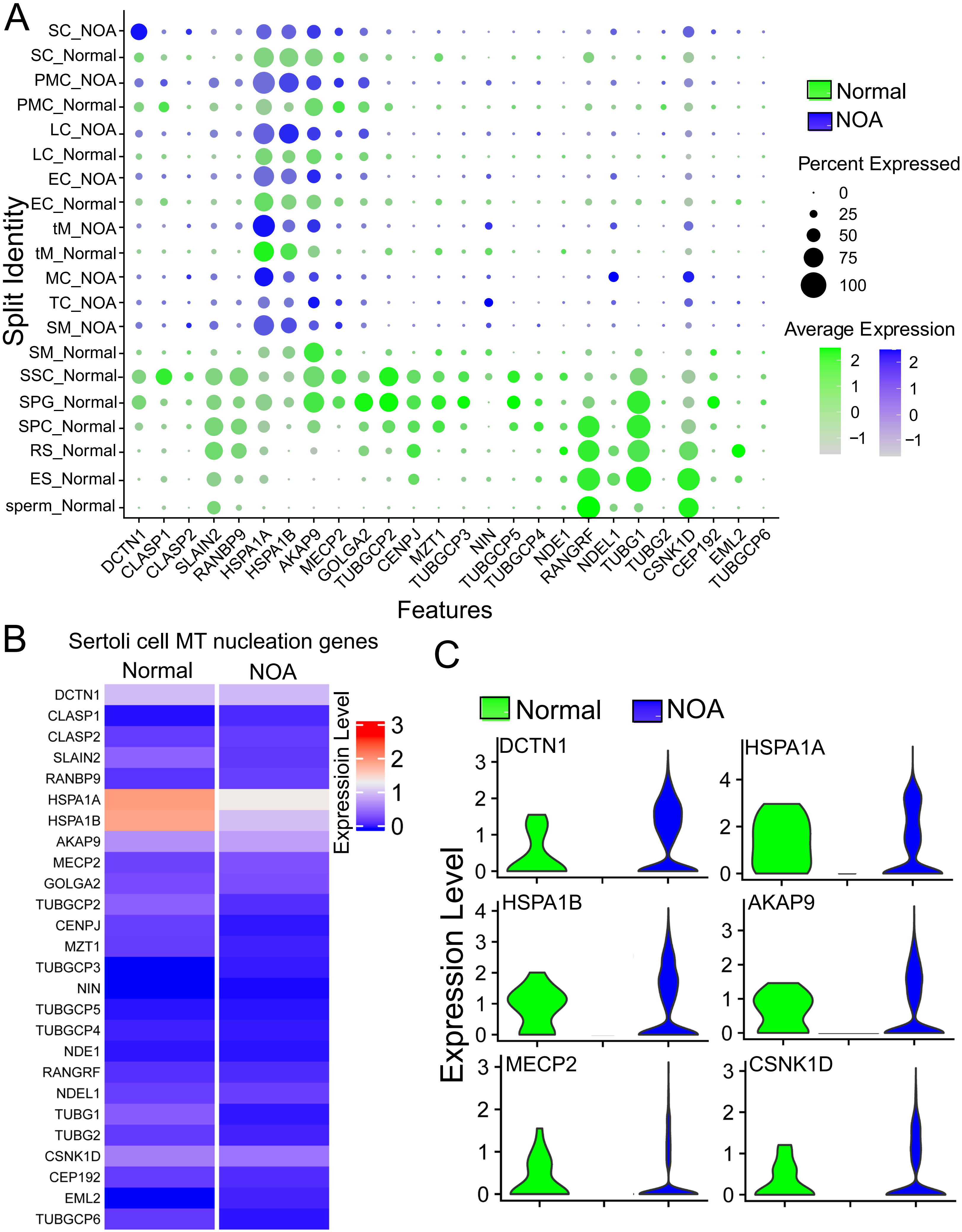

Figure S4 (Wu X et al.)

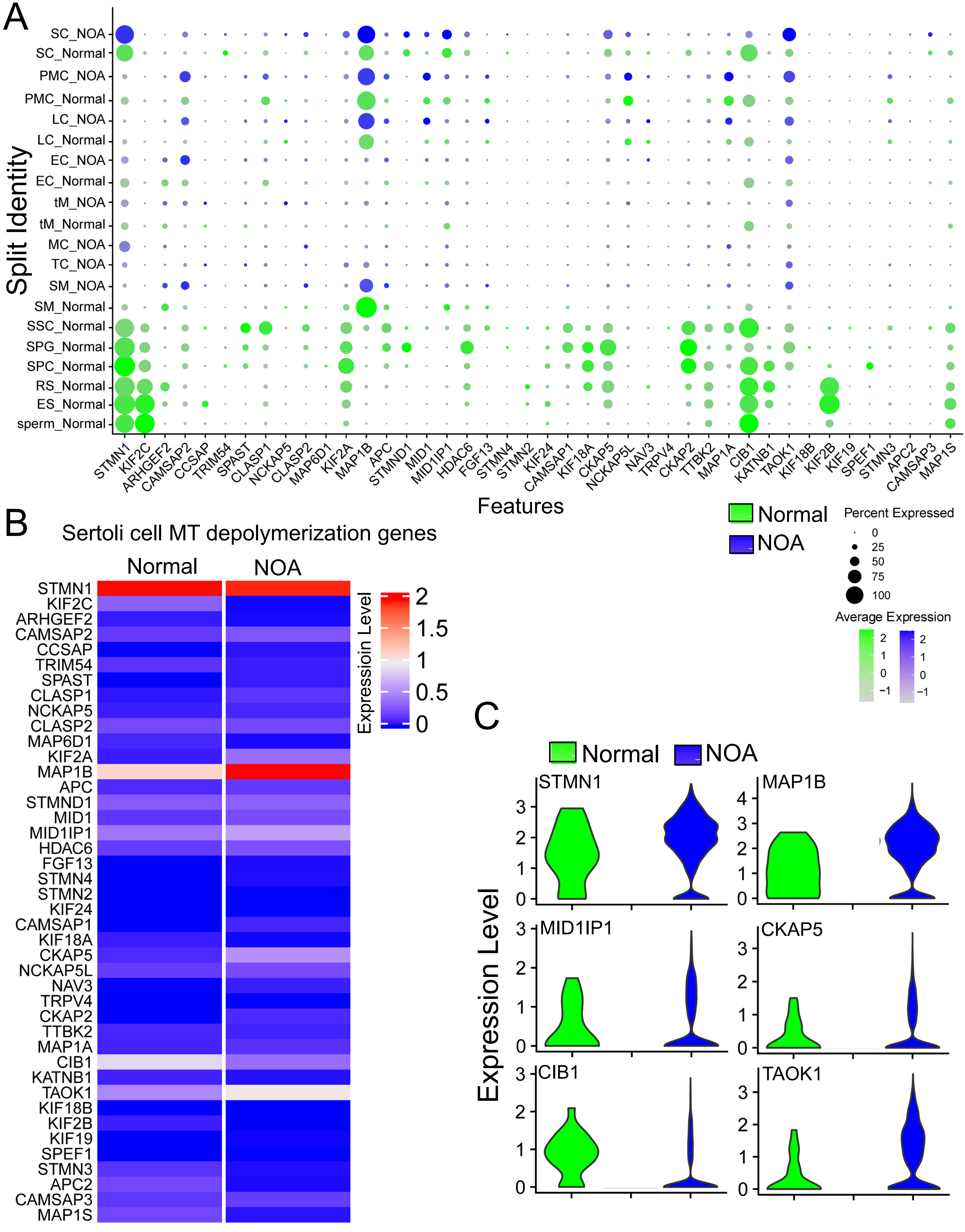

Figure S5 (Wu X et al.)

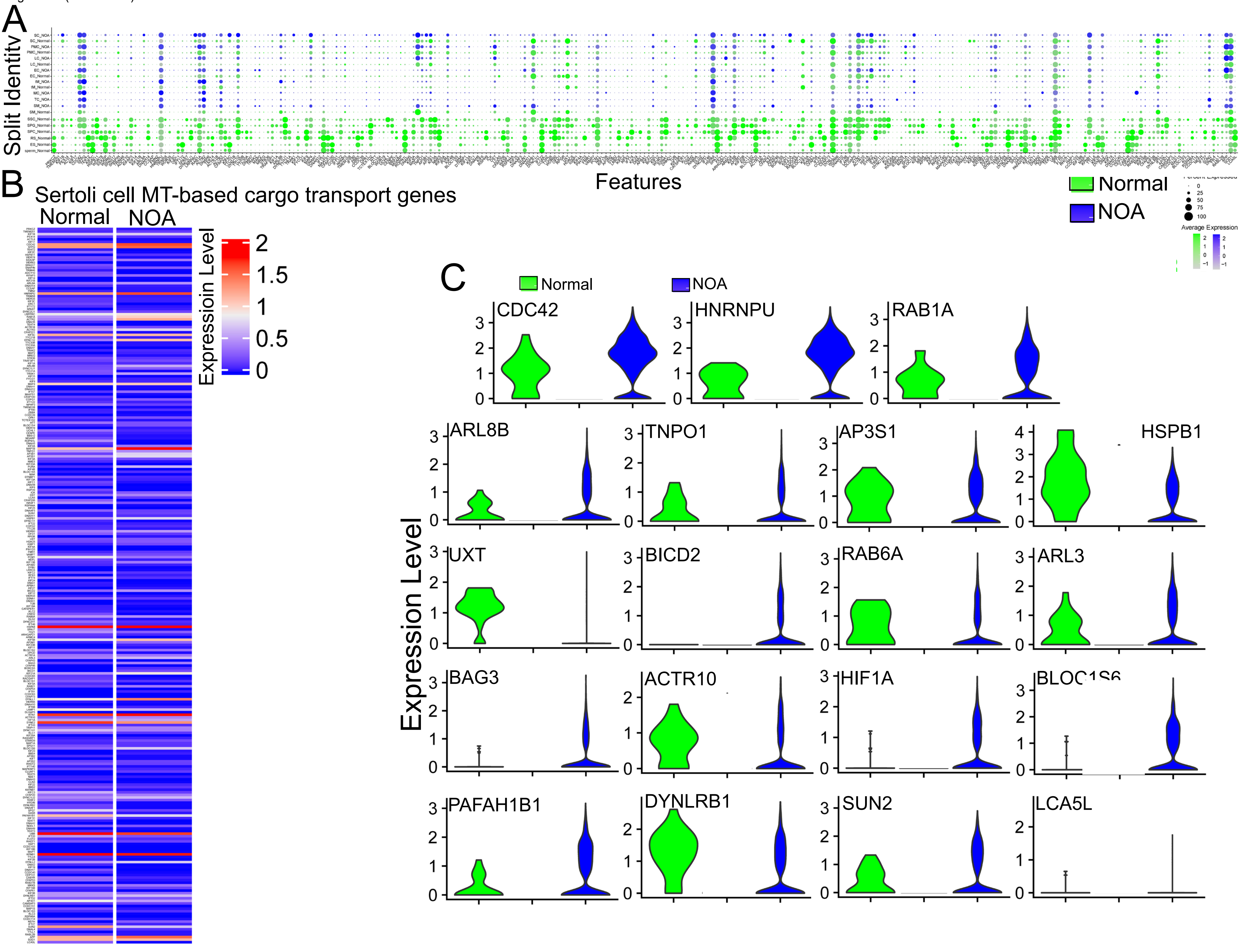

Supplement: Supplementary file 1 — Additional file 1: Figure S1. Differential expression of genes encoding proteins that regulate MT organization in normal vs. OA and NOA human testes based on analyses of scRNA-Seq datasets. (A) scRNA-Seq datasets from normal vs. OA and NOA human testes were analyzed used Seurat package in R toolkit using Rstudio to obtain Dot plot to identify differentially expressed genes encoding genes pertinent to MT organization. It is noted that some of these proteins overlap with proteins examined in Figures S2-S5. Dot size represents the percent (%) expressed, and color depth represents the degree of expression level from 2 to -1. The cell types were Sertoli cells (SC), peritubular myoid cells (PMC), Leydig cells (LC), testicular macrophages (tM), endothelial cells (EC), mast cells, spermatogonia (SPG), round spermatids (RS), and elongated spermatids (ES). (B) Heatmap that illustrate some Sertoli cell differentially regulated genes involved in MT organization in OA and NOA vs. normal testes. (C) Violin plots of some selected Sertoli cell differentially regulated genes involved in MT organization in OA and NOA vs. normal testes. Figure S2. Differential expression of genes encoding proteins involved in microtubule bundling in normal vs. OA and NOA human testes based on analyses of scRNA-Seq datasets. (A) Dot plot of all genes in different testicular cell types that are involved in MT bundling in normal vs. OA nd NOA testes. Dot size represents the percent (%) expressed, and color depth represents the degree of expression level from 2 to -1. The cell types examined herein can be found in Legend to Figure S1. These MT bundling proteins are also involved in mitosis and meiosis to support sister chromatid separation, but also unique structural function such as ectoplasmic specialization (ES) in the testis to support spermatogenesis, as well as structural MAPs (e.g., MAP1a) that confer MT stabilization. For example, MAP1b and MAP1s are involved in the formation of microtubule bundle [file 12958_2022_1026_MOESM1_ESM.pdf]
